# A Step Forward in Molecular Diagnostics of Lyssaviruses – Results of a Ring Trial among European Laboratories

**DOI:** 10.1371/journal.pone.0058372

**Published:** 2013-03-08

**Authors:** Melina Fischer, Kerstin Wernike, Conrad M. Freuling, Thomas Müller, Orhan Aylan, Bernard Brochier, Florence Cliquet, Sonia Vázquez-Morón, Peter Hostnik, Anita Huovilainen, Mats Isaksson, Engbert A. Kooi, Jean Mooney, Mihai Turcitu, Thomas B. Rasmussen, Sandra Revilla-Fernández, Marcin Smreczak, Anthony R. Fooks, Denise A. Marston, Martin Beer, Bernd Hoffmann

**Affiliations:** 1 Institute of Diagnostic Virology, Friedrich-Loeffler-Institut, Greifswald-Insel Riems, Germany; 2 Institute of Molecular Biology, Friedrich-Loeffler-Institut, Greifswald-Insel Riems, Germany; 3 Etlik Central Veterinary Control and Research Institute, Ankara, Turkey; 4 Scientific Institute of Public Health, Brussels, Belgium; 5 Anses Nancy technopole agricole et vétérinaire, Malzeville, France; 6 Instituto de Salud Carlos III; Centro Nacional de Microbiología, Majadahonda, Spain; 7 National Veterinary Institute, Ljubljana, Slovenia; 8 Finnish Food Safety Authority, Helsinki, Finland; 9 Swedish National Veterinary Institute, Uppsala, Sweden; 10 Central Veterinary Institute, Lelystad, The Netherlands; 11 Virology Division, Central Veterinary Research Laboratory, Celbridge, Ireland; 12 Institute for Diagnosis and Animal Health, Bucharest, Romania; 13 National Veterinary Institute, Technical University of Denmark, Lindholm, Denmark; 14 Institute for Veterinary Disease Control, Mödling, Austria; 15 National Veterinary Research Institute, Pulawy, Poland; 16 Animal Health and Veterinary Laboratories Agency, Addlestone, United Kingdom; University of Pretoria, South Africa

## Abstract

Rabies is a lethal and notifiable zoonotic disease for which diagnostics have to meet the highest standards. In recent years, an evolution was especially seen in molecular diagnostics with a wide variety of different detection methods published. Therefore, a first international ring trial specifically designed on the use of reverse transcription polymerase chain reaction (RT-PCR) for detection of lyssavirus genomic RNA was organized. The trial focussed on assessment and comparison of the performance of conventional and real-time assays. In total, 16 European laboratories participated. All participants were asked to investigate a panel of defined lyssavirus RNAs, consisting of *Rabies virus* (RABV) and *European bat lyssavirus* 1 and 2 (EBLV-1 and -2) RNA samples, with systems available in their laboratory.

The ring trial allowed the important conclusion that conventional RT-PCR assays were really robust assays tested with a high concordance between different laboratories and assays. The real-time RT-PCR system by Wakeley et al. (2005) in combination with an intercalating dye, and the combined version by Hoffmann and co-workers (2010) showed good sensitivity for the detection of all RABV samples included in this test panel. Furthermore, all used EBLV-specific assays, real-time RT-PCRs as well as conventional RT-PCR systems, were shown to be suitable for a reliable detection of EBLVs. It has to be mentioned that differences were seen in the performance between both the individual RT-PCR systems and the laboratories. Laboratories which used more than one molecular assay for testing the sample panel always concluded a correct sample result.

Due to the markedly high genetic diversity of lyssaviruses, the application of different assays in diagnostics is needed to achieve a maximum of diagnostic accuracy. To improve the knowledge about the diagnostic performance proficiency testing at an international level is recommended before using lyssavirus molecular diagnostics e.g. for confirmatory testing.

## Introduction

Rabies as a notifiable zoonotic disease is an acute, progressive and incurable viral encephalitis which is clinically characterized by central nervous disorders that ultimately lead to death. The disease is caused by different Lyssavirus species of the *Rhabdoviridae* family [1], with classical rabies virus (RABV) being responsible for tens of thousands of deaths per year [2]. In Europe, alongside sylvatic rabies in foxes, bat rabies is prevalent in a number of different bat species, mainly caused by the European bat lyssaviruses type 1 and 2 (EBLV-1 and 2) [3]. From single rabid bats e.g. West Caucasian bat lyssavirus (WCBV) [4] and Bokeloh bat lyssavirus (BBLV) [5] were isolated.

Whilst ante-mortem testing is only recommended by the World Health Organization (WHO) for rabies suspect human patients, definite rabies diagnosis in both human and animal samples relies on post-mortem laboratory findings. The widely accepted (post-mortem) “gold standard” method of the WHO and the World Organisation for Animal Health (OIE) is the detection of lyssavirus antigen by the fluorescent antibody test (FAT) [6], [7]. For samples from suspected rabid animals with contact to humans or samples with other epidemiological relevance, virus isolation is recommended as the confirmatory test for inconclusive and negative FAT results [2]. Apart from the inevitably fatal outcome of a rabies infection, reliable rabies diagnosis has to meet the highest possible quality standards because in a human case patient management can be optimized and precautions for the nursing staff can be taken. In an animal case laboratory confirmation of rabies via RT-PCR is on the one hand important for the identification of new lyssavirus species. On the other hand if human contacts occurred with this rabid animal then adequate post exposure prophylaxis (PEP) must be initiated.

With the advance of molecular techniques, reverse transcription polymerase chain reaction (RT-PCR) has been increasingly used for amplification of lyssavirus RNA from sample materials. Nowadays, numerous assays are available; for review see Dacheux et al. (2010) [8]. However, to date, RT-PCR and other amplification systems are not recommended for routine post-mortem diagnosis of rabies, but may be used for epidemiological surveys. Laboratories conducting the analysis should have sufficient experience with the techniques in question and should also apply strict quality control procedures [2]. Nevertheless, especially for ante-mortem diagnosis in humans and decomposed brain samples these techniques showed advantages over conventional virological methods and in those cases may be the only option to obtain a definite diagnosis [9]–[11]. In many laboratories, PCR has already been employed as a rapid diagnostic tool in animal rabies diagnosis in addition to the FAT with the aim to allow early termination of initiated PEP in humans. Also PCR offers options for further virus characterization using sequence analysis.

For generic pan-lyssavirus approaches the hemi-nested assay described by Heaton et al. (1997) [12] has been widely used in daily laboratory routine. Few nested RT-PCR protocols were developed for pan-lyssavirus detection, e.g. Echevarría et al. (2001) and Vázquez-Morón et al. (2006) [13], [14]. More recently, RT-PCR systems using fluorogenic probes allowed the detection of sequence-specific templates in real-time. One of the most widely used lyssavirus TaqMan^©^ assays detects and differentiates RABV from EBLV-1 and EBLV-2. Generic primers and species-specific probes were combined within one reaction [15]. Later on, the specificity of the RABV system was improved by changing some nucleotides of the primers and probe [16]. Alternatively, the Wakeley protocol [15] (named R13 by Hoffmann et al., 2010 [17]) was combined with a second set of primers and probe (R14) downstream of R13 on the nucleoprotein gene to broaden the diagnostic range for RABV [17]. It was also shown that using the primers of the Wakeley protocol with SYBR® Green, which eliminates the requirement for probes, and therefore removes possible problems identified with specificity of the RABV-specific probe, allowed a sensitive pan-lyssavirus detection [18] (further assays are mentioned in Table S1 A+B [32]–[41]).

Newly available chemistries, reagents and procedures have improved and standardised the detection techniques leading to higher sensitivities and specificities. Therefore, validated RT-PCR-based tests were proposed as alternative, confirmatory tests also for rabies, and were suggested to be included in the OIE Manual of Diagnostic Tests and Vaccines for Terrestrial Animals [19]. Furthermore, molecular tools have become an important basis for most of the notifiable and/or zoonotic viral diseases, and lyssaviruses should not be an exclusion.

Previous ring trials focussed mainly on classical methods like FAT and virus isolation or included only a very limited panel of brain tissues for the RT-PCR analysis [20]. Here, we provided for the first time a complete report on a ring trial specifically designed for RT-PCR for the detection of lyssavirus genomic RNA, e.g. RABV and EBLV, focussing on an assessment and comparison of the performance of conventional and real-time RT-PCR assays established in different European laboratories.

## Materials and Methods

A panel of 28 lyssavirus samples from the virus archive of the Friedrich-Loeffler-Institut (FLI) was selected for the trial. Either original brain material or tissue culture supernatant after infection of MNA cells was used (Table 1). All isolates had been tested positive by using FAT, rabies tissue culture infection test (RTCIT) and real-time RT-PCRs for RABV, EBLV-1 and EBLV-2 (Hoffmann et al., 2010 [17]; Freuling et al., unpublished). In order to cover the very high genetic diversity of the different RABV strains, the panel consisted of 26 RABV RNA samples from different countries across the world and various isolation years, including one log10 dilution series (L-30, L-06, L-27, L-28), as well as one EBLV-1 (L-18) and EBLV-2 (L-24) RNA sample each (Table 1). The extraction of the viral RNAs was performed at the FLI using the RNeasy® kit (Qiagen, Hilden, Germany) according to the manufacturer’s instructions and stored in RNA-safe buffer (50 ng/µl carrier RNA (poly A homopolymer from Qiagen, Hilden, Germany), 0.05% Tween 20 (Sigma, Munich, Germany) and 0.05% sodium acid (NaN_3_) solution (Sigma, Munich, Germany) in RNase free water) [21]. Furthermore, two negative samples (L-07, L-25) containing water or RNA-safe buffer were added. All samples were transferred into labelled reaction tubes and shipped on dry ice to the 16 participating laboratories (Table S2 A) using polystyrene boxes. To mimic influences of transportation, one sample set was subjected to a freeze-thaw-cycle and subsequently tested by real-time PCR (freeze-thaw control; Table S3). Particular attention was paid to provide the same material to all labs in good condition.

**Table 1:** Data description of the samples (n = 30) included into the lyssavirus panel.

Lab ID	Ring trial number	Virusspecies	Genetic lineage	Year of isolation	Origin	Source	Host	R13*	R14*	R13/14*
12952	L-01	RABV	NEE	2001	Estonia	BS	fox	31.0	27.4	27.8
13091	L-02	RABV	Middle east	1991	Abu Dabi	BS	camel	17.5	16.7	16.7
20280	L-03	RABV	Arctic-like	2006	Afghanistan	BS	dog	15.7	no C_q_	17.0
20295	L-04	RABV	Middle East	2009	Iraq	BS	dog	17.4	12.9	13.4
13250	L-05	RABV	–	1973	Chile	BS	human	no C_q_	28.8	28.8
**20293 10^−1^**	L-06	RABV	Middle East	2008	Iraq	BS	dog	–	–	–
NTC	L-07	–	–	–	–	–	–	–	–	–
13001	L-08	RABV	NEE	1990	Estonia	TCS	raccoon	30.9	27.3	27.7
13136	L-09	RABV	Africa 2	1989	Nigeria	BS	–	22.8	no C_q_	22.9
20281	L-10	RABV	Arctic-like	2006	Afghanistan	BS	dog	14.9	no C_q_	17.3
20297	L-11	RABV	Middle East	2009	Iraq	BS	horse	14.9	11.9	11.8
13254	L-12	RABV	–	1979	Chile	BS	human	no C_q_	20.5	20.5
13213	L-13	RABV	–	1981	USA	TCS	skunk	28.0	25.8	25.2
13044	L-14	RABV	Middle-East	1990	Saudi Arabia	TCS	fox	20.5	19.4	19.5
13138	L-15	RABV	Africa 2	1989	Nigeria	BS	dog	–	–	–
20290	L-16	RABV	Middle East	2008	Iraq	BS	cow	24.2	25.2	24.3
11164	L-17	RABV	CEE	2005	Germany	BS	fox	no C_q_	23.4	23.9
EBLV-1	L-18	EBLV-1	EBLV-1a	1968	Germany	BS	bat	–	–	–
20294	L-19	RABV	Middle East	2008	Iraq	BS	dog	16.6	12.5	12.5
13078	L-20	RABV	EE	1995	Bulgaria	BS	human	13.8	12.7	12.8
13205	L-21	RABV	–	1981	USA	BS	raccoon	–	–	–
20291	L-22	RABV	Middle East	2008	Iraq	BS	dog	22.6	18.5	19.1
SAD-B19	L-23	RABV	vaccine	1991	Germany	TCS	vaccine	18.2	19.0	18.3
EBLV-2	L-24	EBLV-2	EBLV-2a	1985	Finland	BS	human	–	–	–
RSB50	L-25	–	–	–	–	–	–	–	–	–
13081	L-26	RABV	–	1985	China	TCS	–	26.1	23.0	22.7
**20293_10^−2^**	L-27	RABV	Middle East	2008	Iraq	BS	dog	–	–	–
**20293_10^−3^**	L-28	RABV	Middle East	2008	Iraq	BS	dog	–	–	-
13240	L-29	RABV	*Eptesicus fuscus*	1986	Canada	BS	bat	no C_q_	25.4	25.5
**20293**	L-30	RABV	Middle East	2008	Iraq	BS	dog	16.6	12.6	13.0

dilution series is depicted in bold; NTC: no template control; BS: brain suspension; TCS: tissue culture supernatant; NEE: North Eastern Europe; CEE: Central and Eastern Europe; EE: Eastern Europe; RABV: *Rabies virus*; EBLV: *European Bat Lyssavirus*; –: not tested;

* C_q_ values from previous publication [17]; no C_q_: no C_q_ value detected; R13, R14, R13/14: R13, R14, duplex R13/14 assay by [17].

All participants were asked to investigate the complete set of 30 blinded samples in duplicate with the diagnostic PCR assays used in their own laboratory. If possible, differentiation of the most common species (RABV, EBLV-1, EBLV-2) was requested and results, e.g. positive/negative for conventional and C_q_ values for real-time PCRs, respectively, had to be reported using an Excel-spread sheet. Each test was designated to the respective laboratory (designated as A-P), and if two or more methods were performed an additional number (A1, A2, etc.) was added. Furthermore, additional information on the established RT-PCR assays e.g. published or unpublished (in-house) method, RT-PCR kits used and modifications of protocols were also requested and recorded.

## Results

In summary, nine different published and five unpublished (in-house) assays mostly targeting the nucleoprotein gene were used in the frame of this ring trial, including real-time RT-PCR (14 labs) as well as conventional RT-PCR (5 labs) techniques (Tables 2, 3, and 4). These techniques comprised both two-step (n = 2) and one-step (n = 11) RT-PCR systems. In this ring trial eleven laboratories relied on real-time PCR only, whereas two laboratories only used conventional techniques (Table S2 B). Three laboratories used both techniques in parallel. For the viral RNA detection, most laboratories (12 out of 16) performed two or more tests. For real-time RT-PCR detection, different modified protocols (lab-versions) of the assay published by Wakeley et al. (2005) [15] and the assay developed by Hoffmann et al. (2010) [17] were most frequently used (Table 2). A lab-version of the original protocol could differ slightly by changes of the PCR kit chemistry and/or adjustment of the temperature profile. In detail, six laboratories used a lab-version of the Wakeley protocol (R13) alone, while in two laboratories versions of both, the Wakeley (R13) and the Hoffmann protocol (R14) were run separately. In three labs, variations of the combined version (R13/14) were used as a single-tube reaction (Table 2). Additionally, one laboratory also applied the assay published by Orlowska et al. (2008) [22]. This assay is also located in the nucleoprotein encoding gene and uses a nearly identical detection region for RABV as the Wakeley assay (98 nucleotides overlap of the amplified region). Furthermore, an in-house broad spectrum system based on detection via an intercalating dye was used by one laboratory (G).

**Table 2:** Comparative analysis of viral genome detection via real-time RT-PCR. Mean C_q_ values from duplicate runs.

		Broad spectrum approach						RABV-specific												
		i.h.	2)	Wakeley et al., 2005									Hoffmann et al., 2010							
Sample	Species		r, e1	r, e1+2				r (R13)					r (R14)					r (R13/14)		
		int. dye	probes	probes			int. dye	probe					probe					probes		
		G ts	D3	B	C	D1	N1 ts	P1	I^mod.^	M	N2 ts	A2	A1	E	F1	J	P2	D2	F2	O
**L-01**	RABV	32	32	27	28	31	37	26	32	23	34	25	27	25	27	30	25	33	27	25
**L-02**	RABV	24	32	25	27	29	32	24	31	27	32	24	26	23	25	28	23	29	25	24
**L-03**	RABV	32*	*fn*	26	27	30	34	24	32	27	32	24	32	**exp**	**exp**	**exp**	29	30	30	25
**L-04**	RABV	25	*fn*	26	30	30	33	24	33	27	32	23	24	23	24	24	22	29	25	24
**L-05**	RABV	24	26	26	**exp**	**exp**	30	28	**exp**	**exp**	**exp**	**exp**	21	23	20	21	20	27	21	21
**L-06**	RABV (I)	30	*fn*	29	33	33	37	27	37	29	35	26	27	25	27	29	25	32	26	27
**L-07**	neg	na	–	–	–	–	–	–	–	–	–	–	–	–	–	–	–	–	–	–
**L-08**	RABV	29	30	26	27	30	35	25	31	28	34	24	26	24	25	28	24	32	26	25
**L-09**	RABV	33	24	20	24	24	26	29	29	21	24	18	**exp**	**exp**	**exp**	**exp**	**exp**	26	23	20
**L-10**	RABV	33*	31	23	25	28	32	22	28	25	30	22	31	**exp**	**exp**	**exp**	28	29	28	23
**L-11**	RABV	26	30	26	29	30	34	24	32	29	33	23	25	24	24	27	23	29	24	23
**L-12**	RABV	15	21	21	**exp**	**exp**	23	21	**exp**	**exp**	**exp**	**exp**	14	17	15	17	13	20	15	15
**L-13**	RABV	25	24	18	22	23	28	18	25	20	27	17	18	17	18	19	17	23	19	18
**L-14**	RABV	23	*fn*	23	22	27	32	21	31	24	33	20	23	21	23	23	21	28	23	21
**L-15**	RABV	*fn*	33	27	33	32	36	26	31	29	35	24	**exp**	**exp**	**exp**	**exp**	**exp**	33	31	27
**L-16**	RABV	22	31	25	30	30	34	25	33	26	34	24	28	25	27	29	25	32	28	25
**L-17**	RABV	11	24	22	**exp**	**exp**	27	23	39	**exp**	27	22	20	21	21	23	19	27	20	20
**L-18**	EBLV-1	19	25	27	30	27	30	–	–	–	–	–	–	–	–	–	–	–	–	–
**L-19**	RABV	28	30	26	28	29	34	24	33	27	34	23	24	23	24	24	23	29	24	24
**L-20**	RABV	23	24	21	23	29	29	19	26	23	30	20	20	19	18	23	18	26	20	19
**L-21**	RABV	32	*fn*	21	37	*fn*	31	21	28	24	30	21	28	*fn*	*fn*	*fn*	27	*fn*	*fn*	23
**L-22**	RABV	28	36	28	31	31	35	26	34	30	36	25	26	24	26	27	24	30	26	25
**L-23**	RABV	23	22	19	19	23	29	17	21	22	29	17	20	19	20	23	19	24	19	18
**L-24**	EBLV-2	23	–	22	29	25	28	–	–	–	–	–	–	–	–	–	37#	–	–	31#
**L-25**	neg	−(45)	–	–	–	–	–	–	–	–	–	–	–	–	–	–	–	–	–	–
**L-26**	RABV	*fn*	33	25	*fn*	? (40)	? (43)	32	36	34	? (41)	30	27	*fn*	28	*fn*	30	35	27	23
**L-27**	RABV (II)	33	*fn*	33	36	36	? (40)	30	40	35	? (39)	30	31	?	30	30	29	36	29	30
**L-28**	RABV (III)	34*	*fn*	*fn*	39	? (40)	*fn*	34	*fn*	39	*fn*	33	34	*fn*	33	*fn*	32	? (39)	34	36
**L-29**	RABV	25	*fn*	**exp**	**exp**	**exp**	31	**exp**	**exp**	**exp**	**exp**	**exp**	18	20	19	21	17	25	19	19
**L-30**	RABV (0)	24	*fn*	26	29	29	33	24	32	27	33	24	24	23	23	27	22	29	24	24

RABV: *Rabies virus*; ;EBLV: *European Bat Lyssavirus*;neg.: negative control; −: negative result; #: cross-reactivitiy with other *Lyssavirus* species; ?: doubtful result;

* doubtful result was retested; i.h.: in-house assay; dilution series (0), (I), (II), (III); 10^0^, 10^−1^, 10^−2^, 10^−3^; mod.: assay modified; r: RABV-specific detection; e1: EBLV-1 specific; e1+2: EBLV-1+−2 specific; r13, r14, r13/14: R13, R14, duplex R13/14 assay by [17]; *fn*: false negative results; **exp**: expected negative results from previous publication; ts: two-step systems; no duplicates for assays D2, D3 and M; 2) Orlowska et al., 2008 [22].

**Table 3:** Mean quantification cycle (C_q_) values of viral genome detection via EBLV-1 and EBLV-2 specific real-time RT-PCR.

	Participants																		
Species	Freuling unpublished						Wakeley et al., 2005											in-house assay	
	A3	A4	O1	O2	E1*	E2*	F3	F4	I1	I2	J1	L1	L2	M1	M2	N3 ts	N4 ts	P3	P4
RABV	–	–	–	–	–	–	–	–	–	–	–	–	–	–	–	–	–	–	–
RABV	–	–	–	–	–	–	–	–	–	–	–	–	–	–	–	–	–	–	–
RABV	–	–	–	–	–	–	–	–	–	–	–	–	–	–	–	–	–	–	–
RABV	–	–	–	–	–	–	–	–	–	–	–	–	–	–	–	–	–	–	–
RABV	–	–	–	–	–	–	–	–	–	–	–	–	–	–	–	31#	–	–	–
RABV (I)	–	–	–	–	–	–	–	–	–	–	–	–	–	–	–	–	–	–	–
neg	–	–	? (32)	–	–	–	–	–	–	–	–	–	–	–	–	–	–	–	–
RABV	–	–	–	–	–	–	–	–	–	–	–	–	–	–	–	–	–	–	–
RABV	–	–	–	–	–	–	–	–	–	–	–	–	–	–	–	–	–	–	–
RABV	–	–	–	–	–	–	–	–	–	–	–	–	–	–	–	–	–	–	–
RABV	–	–	–	–	–	–	–	–	–	–	–	–	–	–	–	–	–	–	–
RABV	–	–	–	–	–	–	–	–	–	–	–	–	–	–	–	24#	-	–	–
RABV	–	–	–	–	–	–	–	–	–	–	–	–	–	–	–	–	–	–	–
RABV	–	–	–	–	–	–	–	–	–	–	–	–	–	–	–	–	–	–	–
RABV	–	–	? (36)	–	–	–	–	–	–	–	–	–	–	–	–	–	–	–	–
RABV	–	–	–	–	–	–	–	–	–	–	–	–	–	–	–	–	–	–	–
RABV	–	–	–	–	–	–	–	–	–	–	–	–	–	–	–	–	–	–	–
EBLV–1	18	–	21	–	20	–	26	–	28	–	28	24	–	26	–	31	–	20	–
RABV	–	–	–	–	–	–	–	–	–	–	–	–	–	–	–	–	–	–	–
RABV	–	–	–	–	–	–	–	–	–	–	–	–	–	–	–	–	–	–	–
RABV	–	–	–	–	–	–	–	–	–	–	–	–	–	–	–	–	38#	–	–
RABV	–	–	–	–	–	–	–	–	–	–	–	–	–	–	–	–	–	–	–
RABV	–	–	–	–	–	–	–	–	–	–	–	–	–	–	–	–	–	–	–
EBLV–2	–	17	? (13)	21	–	20	–	22	–	28	18	–	25	–	24	–	29	–	17
neg	–	–	? (29)	–	–	–	–	–	–	–	–	–	–	–	–	–	–	–	–
RABV	–	–	–	–	–	–	–	–	–	–	–	–	–	–	–	–	–	–	–
RABV (II)	–	–	–	–	–	–	–	–	–	–	–	–	–	–	–	–	–	–	–
RABV (III)	–	–	? (25)	–	–	–	–	–	–	–	–	–	–	–	–	–	–	–	–
RABV	–	–	–	–	–	–	–	–	–	–	–	–	–	–	–	–	–	–	–
RABV (0)	–	–	–	–	–	–	–	–	–	–	–	–	–	–	–	–	–	–	–

RABV: *Rabies virus*;;EBLV: *European Bat Lyssavirus*;neg.: negative control; –: not applicable; #: cross–reactivitiy with other *Lyssavirus* species; ?: inconclusive based on curve shape; dilution series (0), (I), (II), (III); 10^0^, 10^−1^, 10^−2^, 10^−3^;

* Hoffmann and Müller personal communication; ts: two-step systems; no duplicates for assays M1 and M2; all laboratories except J1 used separate species-specific real-time PCRs to detect EBLV-1 or -2.

**Table 4:** Comparison of conventional PCR systems for the detection of RABV and/or EBLV-1 and EBLV-2.

		Participants									
		V.-M. et al., 2006		Heaton et al., 1997			Ech. et al., 2001			i.h.	M/V
Sample	Species	H^p^	I3^p^	J^p^	K^p,^ *	I5^p^ts	I1^p^	I2^p^	I4^r, 1)^ts	O1^r^	O2^e^
**L-01**	RABV	RABV	+	+	+	+	+	+	*fn*	RABV	na
**L-02**	RABV	RABV	+	+	+	+	+	+	+	RABV	na
**L-03**	RABV	RABV	+	+	+	+	+	+	+	*fn*	−
**L-04**	RABV	RABV	+	+	+	+	+	+	+	RABV	na
**L-05**	RABV	RABV	+	+	+	+	+	+	+	RABV	na
**L-06**	RABV (I)	RABV	+	+	+	+	+	+	+	RABV	na
**L-07**	neg	−	−	−	−	−	−	−	−	−	−
**L-08**	RABV	RABV	+	+	+	+	+	+	+	RABV	na
**L-09**	RABV	RABV	+	+	+	+	+	+	*fn*	RABV	na
**L-10**	RABV	RABV	+	+	+	+	+	+	+	*fn*	-
**L-11**	RABV	RABV	+	+	+	+	+	+	+	RABV	na
**L-12**	RABV	RABV	+	+	+	+	+	+	+	RABV	na
**L-13**	RABV	RABV	+	+	+	+	+	+	+	RABV	na
**L-14**	RABV	RABV	+	+	+	+	+	+	+	RABV	na
**L-15**	RABV	*fn*	*fn*	+	+	+	+	?	+	RABV	na
**L-16**	RABV	RABV	+	+	+	+	+	+	+	RABV	na
**L-17**	RABV	RABV	+	+	+	*fn*	+	+	+	RABV	na
**L-18**	EBLV-1	EBLV1a	+	+	+	+	+	+	−	−	EBLV-1
**L-19**	RABV	RABV	+	+	+	+	+	+	*fn*	RABV	na
**L-20**	RABV	RABV	+	+	+	+	+	+	+	RABV	na
**L-21**	RABV	RABV	+	+	+	+	?	+	+	*fn*	−
**L-22**	RABV	RABV	+	+	+	+	+	+	+	RABV	na
**L-23**	RABV	RABV	+	+	+	+	+	+	+	RABV	na
**L-24**	EBLV-2	EBLV2a	+	+	+	+	+	+	−	−	EBLV-2
**L-25**	neg	−	−	**fp**	−	−	−	−	−	−	−
**L-26**	RABV	RABV	+	+	+	+	+	+	+	RABV	na
**L-27**	RABV (II)	RABV	+	+	+	+	+	+	+	RABV	na
**L-28**	RABV (III)	RABV	+	*fn*	*fn*	+	+	+	*fn*	*fn*	−
**L-29**	RABV	RABV	+	+	+	+	+	+	+	*fn*	−
**L-30**	RABV (0)	RABV	+	+	+	+	+	+	*fn*	RABV	na

RABV: *Rabies virus*; ;EBLV: *European Bat Lyssavirus*;neg.: negative control; +: positive genome detection; -: negative result; ?: doubtful result; i.h.: in-house assay; na: not applicable; dilution series (0), (I), (II), (III); 10^0^, 10^−1^, 10^−2^, 10^−3^; p: detection of RABV, EBLV-1 and -2; r: RABV-specific detection; e: EBLV-specific; **fp**: false positive result; *fn*: false negative results; ts: two-step system;

* Bourhy unpublished data; Ech. et al., 2001: [13]; M/V: [26] and [27]; V.-M. et al., 2006: [14]; 1) Ito et al., 2001 [25].

To rule out variability as a result of RNA extraction methodologies, RNA was extracted prior to the shipment to the participating laboratories. To maintain RNA stability during transportation, samples were stored in RNA-safe buffer [21] and shipped on dry ice. In order to confirm RNA stability after suboptimal transportation, one sample set was subjected to a freeze-thaw-cycle. Subsequent real-time PCR testing of this freeze-thaw control did not reveal any noticeable increase of C_q_ values (Table S3).

### Real-time RT-PCR

The results of the real-time RT-PCR genome detection in the different laboratories are summarized in Tables 2 and 3. The individual laboratories used one to five microliter template RNA for their real-time PCR investigations. None of the applied real-time RT-PCR systems or runs produced false-positive results for the negative samples but two EBLV-2 samples scored positive in RABV-specific real-time PCRs (P2, O). Detection of viral RNA via RABV-specific or pan-lyssavirus real-time RT-PCR often failed for single RABV isolates (Table 2), while ten of the 30 samples (L-1, L-2, L-8, L-11, L-13, L-16, L-19, L-20, L-22 and L-23) were always correctly detected. Some participating laboratories failed to identify certain isolates, while others obtained a positive result using lab-versions of the same published protocol (Table 2). The system by Wakeley et al. (2005) [15] used generic primers either with a RABV-specific or with EBLV-1- and -2-specific hydrolysis probes described here as broad spectrum approach. Additionally, one lab (N1) also used a two-step modification of the Wakeley protocol with an intercalating dye instead of hydrolysis probes for fluorescence detection, based on Hayman et al. (2011) [23]. This lab-version was able to detect all positive lyssavirus samples correctly with exception of the 1.00E−3 dilution step of RABV isolate 20293 (L-28). Furthermore, two results were reported as doubtful which would require further investigations.

Taking the best scoring laboratory results for each assay (P1 for R13, A1 and P2 for R14, O for R13/14) a combination of R13 and R14 assays (in single wells or as duplex assay) were able to detect all rabies samples very robustly. However, sample L-29 was not detected by any of the applied R13 assays and samples L-05, L-12 and L-17 were unreliably detected. Samples L-09 and L-15 from Nigeria (clade Africa 2) were not detected by the R14 system (described previously by [17]) while samples L-03, L-11 and L-21 were unreliably detected. Moreover, some samples were unreliably detected by both assays; specifically L-03, L-09, L-10, L-15, L-21 for R14 and L-05, L-12, L-17 and L-29 for R13. Interestingly, by combining the results from laboratories A and P where R13 and R14 versions were applied in parallel, all positive samples were recognized correctly with a similar sensitivity to the duplex assay.

The results from laboratories O (R13/14) and P2 (R14) for sample L-24 provide some indication for cross-reactivity of these rabies-specific methods with EBLV-2. Although this is not a diagnostic disadvantage, as the assay detected a positive lyssavirus sample correctly, it highlights the issues with the use of hydrolysis probes to differentiate *Lyssavirus* species.

Varying the R14 single assay with different PCR chemistries, laboratories E and J produced identical seven false-negative results. Using this assay with the same RT-PCR kit and the same PCR cycler model but slightly different temperature profiles, laboratory P (P2) yielded a better performance with lower C_q_ values (1–3 cycles; except for the Chinese strain L-26) compared to laboratory A (A1). Also, the variation of the C_q_ values for individual samples between different laboratories using a lab-version of the same assay was remarkably broad in several cases.

The assay published by Orlowska et al. (2008) [22] correctly recognized EBLV-1 but failed to detect several RABV isolates including the complete dilution series. Considering the retesting of doubtful results, the two-step in-house assay applied by laboratory G recognized 24 out of the 26 RABV isolates as well as the EBLV-1 and EBLV-2 samples.

In general, EBLV-1 and -2 were recognized correctly by the appropriate assays (Table 3). Only one laboratory (N3+4) obtained a cross-reactivity with some RABV strains using a two-step variant of the Wakeley assay [15], whereas the remaining five laboratories had no problems applying their lab-versions of the assay. Another laboratory obtained some inconclusive results for EBLV-1 detection according to the curve shape by usage of a yet unpublished assay (Freuling, unpublished data). Nevertheless, this unpublished assay provided in laboratory A very robust C_q_ values for the recognition of EBLV-1.

### Conventional RT-PCR

The performance of five conventional RT-PCR methods for RABV-specific detection or a broad range application is shown in Table 4. Although most samples were correctly diagnosed, the 1000 fold dilution step of RABV isolate 20293 (L-28) was not detected by four of the nine conducted runs. Furthermore, at least one sample was not recognized by each of the applied methods or classified as doubtful. Variations of the pan-lyssavirus assays established by Heaton et al. (1997) [12], Echevarría et al. (2001) [13], and Vázquez-Morón et al. (2006) [14] were able to detect almost every RABV and EBLV isolate in this panel (Table 4). Both, the original protocol (lab H) developed by Vázquez-Morón and co-workers (2006) [14] and a lab-version of laboratory I (I5) generally failed to recognize the Nigerian sample L-15. In addition, laboratory H obtained a doubtful result for the 1.00E−3 dilution step of RABV isolate 20293 (L-28). Using a one-step version of the Heaton assay [12], laboratory J (J2) obtained one false-positive result.

Laboratory I (I7) recognized all isolates except the European sample L-17 with a two-step version of this assay. Lab-versions of the system by Echevarría and co-workers (2001) [13] were able to detect all isolates, with the exception of one doubtful result in each run. Two RABV-specific assays (in-house assay based on East et al., 2001 [24]; Ito et al., 2001 [25]) applied by laboratory O and I (O3, I6), were not able to detect five RABV isolates, respectively. Also EBLV-specific conventional RT-PCR assays [26], [27] reliably detected EBLV-1 and -2 (Table 4).

In general, usage of different tests in parallel led to correct or at least questionable results which would require further investigations (Table S4). There are only a few exceptions (samples L-21 and L-28) where parallel testing provided an overall false result. Due to an overall insufficient analytical sensitivity, two laboratories (J, N) were not able to detect the 1000 fold dilution step of RABV isolate 20293 (L-28) using two different tests, respectively. It is more critical that in the hands of laboratories D and F an inadequate degree of diagnostic sensitivity led to a detection failure for sample L-21 despite of a significant viral load.

## Discussion

During the recent validation of a fluorogenic probe-based real-time RT-PCR for RABV, it had become evident, that a single assay was not sufficient to detect all tested RABV strains due to a high degree of genetic diversity [17]. As a consequence, we tried for the first time to assess how published or in-house molecular assays are performing at different European laboratories in an international ring trial.

The results described and discussed here revealed a relatively high degree of divergence among the participating laboratories. Partially, this can be explained by the diagnostic range of the applied assays and has been highlighted in Table 2 for the R13 and R14 assay. To differentiate between a failure of the system due to a test specificity issue according to mismatches in the primer and/or probe region (expected negative; Hoffmann et al., 2010 [17]) or due to laboratory discrepancies (false negative), different labellings were used. Interestingly, some samples tested positive in one laboratory and were not detected in the other. Various reasons could be responsible for such an unexpected pattern, e.g. quality of extracted RNA (degradation), primers, probes, PCR machines and used commercial real-time RT-PCR kits. In this case the use of an internal control system could be helpful to elucidate this finding.

In previous European ring trials for rabies routine diagnosis [20] both conventional techniques, e.g. FAT and rabies tissue culture infection test (RTCIT), and real-time RT-PCR had been used on brain tissues [20]. However, the diversity of lyssavirus isolates was very limited and therefore, results are not easily comparable. In contrast, our study was solely dedicated to assessing established RT-PCR assays for RABV in particular. This might explain why most laboratories (n = 11; Table S2 B) performed real-time PCR exclusively. Alternatively, brain homogenates would be a suitable option for the next ring trial, enabling the application of internal control assays such as β-actin, which will aid interpretation of the negative results, already a vital element in any diagnostic assay.

Ring trials as the one described here trigger diagnostic laboratories to start intensive investigations on the diagnostic quality, so that an overall improvement can be made.

A first problem that may be associated with comparing the performance of RT-PCR assays is the stability of the RNA during both, transport and testing. In our case, RNA degradation is unlikely as the RNA was preserved in a special storage buffer and shipment was done on dry ice. Furthermore, a freeze-thaw control was used to confirm RNA stability. A similar approach proved to be successful for a recent European classical swine fever virus (CSFV) ring trial [28]. In the future, samples could also be spiked with an internal control, e.g. EGFP [21] to allow for monitoring of PCR performance. There is proven evidence that commercially available RT-PCR test kits perform differently and can have a substantial impact on the RT-PCR results obtained [29].

As a main conclusion the R13/14 RABV-specific real-time RT-PCR system [17], used as a duplex assay or in combination of both single assays, displayed the best sensitivity for RABV detection among all applied real-time RT-PCR assays during this ring trial. The Wakeley assay [15] performed by lab B also displayed good results by detecting all RABVs (apart from the known issue with L-29 and the 1.00E−3 dilution (L-28)). Also, both EBLV-1 and -2 were correctly detected with the specific primers and probes. The application of this broad spectrum assay with an intercalating dye (N1, two-step) enabled the detection of L-29 whilst also detecting all other samples correctly, except the 1.00E−3 dilution (L-28) and two doubtful results. It is likely that the diluted sample was not detected due to the application of a two-step methodology because only a proportion of the cDNA is used in the two-step assay. In comparison to a two-step system all the available cDNA can be used in the one-step/one-tube system.

In North America, TaqMan® PCR assays for the detection of RABV were either comparable, or they had a considerably reduced detection limit compared to hemi-nested PCR [30], [31]. In contrast, in this ring trial, the panel of RABV strains included was of such genetic heterogeneity, that particularly the hydrolysis-based assays displayed problems in detecting certain strains, mostly not belonging to the cosmopolitan variant. Therefore, in order to overcome these problems, the parallel use of several (real-time) RT-PCR assays in a diagnostic setting is highly recommended.

One argument against PCR diagnostics in the field of rabies, but also in general, is the risk of contamination leading to false-positive results. However, carry-over contamination from positive controls can be prevented efficiently by strict quality control procedures in place, such as using artificial positive controls [17]. In the context of this ring trial, false-positive results were not observed, indicating high laboratory quality standards. However, we only asked for testing extracted RNA, and therefore the RNA extraction step could not be evaluated, both in terms of sensitivity as well as carry-over contamination. False-negative results due to PCR inhibition are also critical. This possibility can easily be ruled out by the use of an internal control as performed by five of the 16 laboratories using β-actin or external heterologous control systems.

Most conventional RT-PCR methods performed satisfactorily for RABV detection and in general were less error-prone; nearly all samples were recognized, than the real-time PCR approaches. Also one conventional RT-PCR assay generally failed to detect a certain isolate (L-15) presumably according to mismatches in the primer binding region due to viral diversity. Furthermore, there were fewer inter-laboratory variations when individual versions of the same assay were used. One possible explanation could be that conventional RT-PCRs are well established in many rabies diagnostic laboratories, whereas the implementation of real-time RT-PCRs is an on-going process. This is a very important point, as we would predict a similar situation with the real-time assays as they have become more embedded and routinely applied. While real-time RT-PCR assays could be used for rapid rabies diagnosis, conventional RT-PCR methods will remain valuable since sequence information can be obtained for subsequent phylogenetic analysis. Furthermore, all applied EBLV-specific investigations, real-time as well as conventional systems were suitable for a reliable EBLV diagnosis, although additional studies are required, since only single samples of each virus has been included into the study panel.

Based on molecular techniques using any kind of PCR diagnostics, 29 inconclusive or false negative results occurred resulting in an overall sensitivity of 93% (70.0–100%) for RT-PCR. Although this may be acceptable, in total, only four of the 16 laboratories submitted 100% concordant results for RABV diagnosis. This appears to be a very low proportion, considering that during rabies diagnostic ring trials 90.5% and 80.5% of participating laboratories produced satisfactory results with no false negative results in 2009 and one false negative result in 2010, respectively [20]. In these previous ring trials false positive results occurred only in laboratories were also nested PCR was performed, presumably in consequence of cross-contamination. Furthermore, in previous ring trials the panel consisted of very few RABV strains. The sample material consisted of brain tissue homogenates and no dilution series was included. In general, the analysis of the dilution series revealed differences in sensitivity between the various approaches. Altogether, the serial dilution of RABV isolate 20293 was detected correctly by only 53% (16 out of 30 tests) of the appropriate real-time and conventional RT-PCR investigations. In another 30% (9 out of 30 runs) of cases all dilution steps except the 1.00E−3 dilution could be recognized. Although a 1.00E−3 dilution does not seem very critical for a highly sensitive method, the initial undiluted sample already had C_q_ values in the high twenties, indicating a low amount of viral RNA. Thus by diluting further, some assays reached their diagnostic limits in terms of sensitivity. However, one has to keep in mind that false-negative results would be a major problem in animal rabies diagnosis where a human exposure occurred because this can cause human fatality. Thus, parallel testing is again recommended in this case.

The overall analysis of the ring trial showed that RT-PCR could be a very reliable diagnostic tool if assays with the broadest diagnostic range are used and quality standards are met at each level. Then, RT-PCR methods are probably suitable to be used in a qualified and trained laboratory as a second diagnostic line in parallel to traditional methods like FAT, mouse inoculation test (MIT) and RTCIT. For this purpose, a further harmonisation and standardisation of the individual methods e.g. by the use of commercial RT-PCR kits as seen in the case of CSFV detection [28] or RT-PCR trainings could help to improve the overall performance between the individual laboratories. Moreover, the ability to sequence an RT-PCR amplicon is extremely useful for surveillance purposes by determining and characterising the Lyssavirus species detected.

Here, we used a very broad and complex RNA panel to test the assays used as much as possible. Nevertheless, this ring trial was not intended to discredit any methodology. However, if a laboratory involved in rabies control uses a certain established protocol that solely recognizes the prevailing RABV variants, problems can occur if a case of imported rabies occurs. In this case, the molecular diagnosis needs to be as broad as possible. This is particularly important if laboratories act as national reference laboratories and are confronted with human rabies diagnosis. In this case, the use of broad spectrum lyssavirus assays may be more suitable, as no prior epidemiological information is necessary. These reference laboratories should particularly be interested in the realisation of further ring trials.

At the moment, neither the WHO nor the OIE have approved molecular techniques for rabies. However, validated PCR-based investigations are proposed to be included in the OIE Manual of Diagnostic Tests and Vaccines for Terrestrial Animals [19]. Based on the results obtained in this study, instead of recommending or approving a single assay, we would finally propose that a proficiency test including a similarly broad standard panel of RABV and indeed other Lyssavirus species should be passed, as performed annually through a program coordinated by the French Agency for Food, Environmental and Occupational Health & Safety (ANSES) [20], before a laboratory is qualified to use RT-PCR as a complementary test in rabies routine diagnosis.

## Supporting Information

Table S1 **Published conventional PCRs for lyssavirus detection and Published real-time PCR assays for lyssavirus detection.**
(DOC)

Table S2 **Participants of the lyssavirus ring trial and Overview of applied RT-PCR techniques and chemistries.**
(DOC)

Table S3 **Mean quantification cycle (Cq) values of a sample set testing before and after a freeze-thaw cycle (Freeze-thaw control).**
(DOC)

Table S4 **Overview of the overall results of laboratories which performed more than one assay.**
(DOC)
