# Supplementary material for: A Step Forward in Molecular Diagnostics of Lyssaviruses – Results of a Ring Trial among European Laboratories
Source: PLoS One. 2013 Mar 8;8(3):e58372. doi: 10.1371/journal.pone.0058372 (PMC3592807; doi:10.1371/journal.pone.0058372)
Supplement: Table S1 — Published conventional PCRs for lyssavirus detection and Published real-time PCR assays for lyssavirus detection. (DOC) [file pone.0058372.s001.doc]

**Table S1 A: Published conventional PCRs for lyssavirus detection**

| **Literature** | **Detected species** | **Type of assay** | **Amplified region** | **Forward primer (5’-3’)** | **Reverse primer (5’-3’)** |
| --- | --- | --- | --- | --- | --- |
| **Biswal et al., 2012 [32]** | RABV | nested RT-PCR | N gene | 1) CTA CAA TGG ATG CCG AC  2) GAC ATG TCC GGA AGA CTGG | 1) GAG TCA CTC GAA TAT GTC  2) GTA TTG CCT CTC TAG CGG TG |
| **De Benedictis et al., 2011 [33]** | RABV, LBV, MOKV, DUVV, EBLV-1+2, ABLV | pyro-sequencing | N gene | AAC ACY YCT ACA ATG GA | TCC AAT TNG CAC ACA TTT TGT G  TCC ART TAG CGC ACA TYT TAT G  TCC AGT TGG CRC ACA TCT TRT G |
| **Dacheux et al., 2008 [34]** | RABV, LBV, MOKV, DUVV, EBLV-1, ABLV | hn RT-PCR | L gene | ATG ACA GAC AAY YTG AAC AA | 1) GGT CTG ATC TRT CWG ARY AAT A  2) TGA CCA TTC CAR CAR GTN G |
| **Vazquez-Moron et al., 2006 [14]** | RABV, LBV, MOKV, DUVV, EBLV-1+2, ABLV | nested RT-PCR | N gene | 1) AAR ATN GTR GAR CAY CAC AC  2) AAR ATG TGY GCI AAY TGG AG | 1) GCR TTS GAN GAR TAA GGA GA  2) TCY TGH CCI GGC TCR AAC AT |
| **Picard-Meyer et al., 2004 [35]** | EBLV-1 | hn RT-PCR | N gene | ATG TAA CAC CYC TAC AAT G | 1) CAR TTV GCR CAC ATY TTR TG  2) GTC CCG AGT GAG ATC TTG A |
| **Ito et al., 2001 [25]** | RABV | RT-PCR | N gene | CTA CAA TGG ATG CCG ACA AGA | CCC ATA TAA CAT CCA ACA AAG TG |
| **Ito et al., 2001 [25]** | RABV | RT-PCR | N gene | ACA GAC AGC GTC AAT TGC AAA GC | CTA GGA TTG ACA AAG ATT TTG CTC |
| **Heaton et al., 1997 [12]** | RABV, LBV, MOKV, DUVV, EBLV-1+2 | hn RT-PCR | N gene | ATG TAA CAC CYC TAC AAT TG | 1) CAA TTC GCA CAC ATT TTG TG  1) CAG TTG GCA CAC ATC TTG TG  1) CAG TTA GCG CAC ATC TTA TG |
| **Heaton et al., 1997 [12]** | RABV, LBV, MOKV, DUVV, EBLV-1+2 | hn RT-PCR | N gene | ATG TAA CAC CYC TAC AAT TG | 2) GTC ATC AAA GTG TGR TGC TC  2) GTC ATC AAT GTG TGR TGT TC  2) GTC ATT AGA GTA TGG TGT TC |

1) primary amplification; 2) second round amplification

*Rabies virus* (RABV), *Lagos bat virus* (LBV), *Mokola virus* (MOKV), *Duvenhage virus* (DUVV), *European bat lyssavirus* type 1 and 2 (EBLV-1, EBLV-2)

**Table S1 B: Published real-time PCR assays for lyssavirus detection**

| **Literature** | **Detected species** | **Type of assay** | **Amplified region** | **Forward primer (5’-3’)** | **Reverse primer (5’-3’)** | **Probe sequence (5’-3’)** |
| --- | --- | --- | --- | --- | --- | --- |
| **Wacharapluesadee et al., 2012 [36]** | RABV | Taqman | N gene | CTG GCA GAC GAC GGA ACC | CAT GAT TCG AGT ATA GAC AGC C | FAM-TCA ATT CTG ATG ACG AGG ATT ACT TCT CCG G-TAMRA |
| **Hayman et al., 2011 [18]** | all species | SYBRGreen | N gene | ATG TAA CAC CYC TAC AAT G | GCA GGG TAY TTR TAC TCA TA |  |
| **Hoffmann et al., 2010 [17]** | RABV | Taqman | N gene | GAT CCT GAT GAY GTA TGT TCC TA | RGA TTC CGT AGC TRG TCC A | FAM-CAG CAA TGC AGT TYT TTG AGG GGA C-TAMRA |
| **Coertse et al., 2010 [37]** | RABV, LBV, MOKV, DUVV | Taqman | N gene | CAC MGS NAA YTA YAA RAC NAA | GTR CTC CAR TTA GCR CAC AT | FAM-CAT CAC ACC TTG ATG ACA ACT CAC AA-BHQ-1 |
| **Nadin-Davis et al., 2009 [16]** | RABV | Taqman | N gene | ATG TAA CAC CYC TAC AAT G | AAT CCA GAG GCT CAT CCT GGT | FAM-CGC GTA GAA CTG TGA CAA CAA CGC TGA-TAMRA |
| **Nadin-Davis et al., 2009 [16]** | RABV | Taqman | N gene | TRA TGA CAA CYC ACA ARA TGT | TCA GTC GCT AGA GGA AAA TGG |  |
| **Nadin-Davis et al., 2009 [16]** | RABV | Taqman | N gene | AYT TCT TCC AYA ARA ACT TYG A |  |  |
| **Orlowska et al., 2008 [22]** | RABV | Taqman | N gene | TAC AAT GGA TGC CGA CAA GA | CAA ATC TTT GAT GGC AGG GTA | FAM-TCA GGT GGT CTC TTT GAA GCC TGA GA-TAMRA |
| **Orlowska et al., 2008 [22]** | EBLV-1 | Taqman | N gene | GAT CCC GAT TTG AAA ACA GC | AGA CCA TGG CTC CAG CTA AA | HEX-GGG ATG AAT GCT GCT AAA TTA GAC CCA-TAMRA |
| **Wacharapluesadee et al., 2008 [38]** | RABV | Taqman | N gene | CTG GCA GAC GAC GGA ACC | CAT CCR ACA AAG TGR ATG AG | FAM-TGY CCY GGC TCR AAC ATY CTY CTT AT-BHQ1 |
| **Saengseesom et al., 2007 [39]** | RABV | SYBRGreen | N gene | 1) GAC ATG TCC GGA AGA CTG G | 1) GTA TTG CCT CTC TAG CGG TG |  |
| **Saengseesom et al., 2007 [39]** | RABV | SYBRGreen | N gene | 2) GTA ACA CCT CTA CAA TGG ATG C | 2) TCA AAT CTT TGA TGG CAG GGT A |  |
| **Nagaraj et al., 2006 [40]** | RABV | SYBRGreen | N gene | CTA CAA TGG ATG CCG AC | CCT AGA GTT ATA CAG GGC T |  |
| **Wakeley et al., 2005 [15]** | RABV | Taqman | N gene | ATG TAA CAC CYC TAC AAT G | GCA GGG TAY TTR TAC TCA TA | FAM- ACA AGA TTG TAT TCA AAG TCA ATA ATC AG -TAMRA |
| **Wakeley et al., 2005 [15]** | EBLV-1 | Taqman | N gene |  |  | HEX- AAC ARG GTT GTT TTY AAG GTC CAT AA-BHQ1 |
| **Wakeley et al., 2005 [15]** | EBLV-2 | Taqman | N gene |  |  | Cy5- ACA RAA TTG TCT TCA ARG TCC ATA ATC AG –BHQ2 |
| **Black et al., 2002 [41]** | RABV | Taqman | N gene | GAT CAR TAT GAG TAY AAA TAT CC | CAA TTC CGA CAC ATT TTG TG | FAM- CCC AAT TCC CTT CTA CAT CAG TAC GT-TAMRA |
| **Black et al., 2002 [41]** | RABV | Taqman | N gene |  | CAG TTA GCG CAC ATC TTA TG | FAM- CCC AGT TCC CTT CTA CAT CAG TAC GT-TAMRA |
| **Black et al., 2002 [41]** | RABV | Taqman | N gene |  | CAG TTG GCA CAC ATC TTG TG | FAM- CCC AAT TTC CTT CTA CAT CAG TAC GT-TAMRA |
| **Black et al., 2002 [41]** | LBV | Taqman | N gene |  |  | FAM- ACA GAT GGG AAG AAA CCT GGT-TAMRA |
| **Black et al., 2002 [41]** | MOKV | Taqman | N gene |  |  | FAM- TAG ATG GAA AGA AAC CAG GGA TAA C-TAMRA |
| **Black et al., 2002 [41]** | DUVV | Taqman | N gene |  |  | FAM- TGT GTG TCC CGA AGA TTG GGT T-TAMRA |
| **Black et al., 2002 [41]** | EBLV-1 | Taqman | N gene |  |  | FAM- TTT ACG TGG ACG CAT GGT CTT GT-TAMRA |
| **Black et al., 2002 [41]** | EBLV-2 | Taqman | N gene |  |  | FAM- AGA GCT ACG GGA TTC TCA TTG CT-TAMRA |

*Rabies virus* (RABV), *Lagos bat virus* (LBV), *Mokola virus* (MOKV), *Duvenhage virus* (DUVV), *European bat lyssavirus* type 1 and 2 (EBLV-1, EBLV-2)

1) first round RT-PCR 2) second round PCR
