# Supplementary material for: A Step Forward in Molecular Diagnostics of Lyssaviruses – Results of a Ring Trial among European Laboratories
Source: PLoS One. 2013 Mar 8;8(3):e58372. doi: 10.1371/journal.pone.0058372 (PMC3592807; doi:10.1371/journal.pone.0058372)
Supplement: Table S2 — Participants of the lyssavirus ring trial and Overview of applied RT-PCR techniques and chemistries. (DOC) [file pone.0058372.s002.doc]

**Table S2 A:** Participants of the lyssavirus ring trial

| **Persons responsible** | **Affiliation** | **Country** |
| --- | --- | --- |
| Bernd Hoffmann | Friedrich-Loeffler-Institut | Germany |
| Thomas Müller, Conrad M. Freuling | Friedrich-Loeffler-Institut | Germany |
| Orhan Aylan | Etlik Central Veterinary Control and Research Institute | Turkey |
| Bernard Brochier | Scientific Institute of Public Health | Belgium |
| Florence Cliquet | Anses Nancy technopole agricole et vétérinaire | France |
| Sonia Vázquez-Morón | Instituto de Salud Carlos III | Spain |
| Peter Hostnik | National Veterinary Institute | Slovenia |
| Anita Huovilainen | Finnish Food Safety Authority | Finland |
| Mats Isaksson | Swedish National Veterinary Institute | Sweden |
| Engbert A. Kooi | Central Veterinary Institute | The Netherlands |
| Jean Mooney | Central Veterinary Research Laboratory | Ireland |
| Mihai Turcitu | Institute for Diagnosis and Animal Health | Romania |
| Thomas B. Rasmussen | National Veterinary Institute | Denmark |
| Sandra Revilla-Fernández | Institute for Veterinary Disease Control | Austria |
| Marcin Smreczak | National Veterinary Research Institute | Poland |
| Anthony R. Fooks, Denise A. Marston | Animal Health and Veterinary Laboratories Agency | United Kingdom |

**Table S2 B:** Overview of applied RT-PCR techniques and chemistries

| Technique used | Conventional RT-PCR | Both conventional and real-time RT-PCR | Real-time RT-PCR |
| --- | --- | --- | --- |
| n laboratories | 2 | 3 | 11 |
| n laboratories with discordant results | 2 | 2 | 9 |
| One-step or two-step RT-PCR | One-step | Both one-step and two-step | Two-step |
| n laboratories | 13 | 1 | 2 |
| n laboratories with discordant results | 9 | 1 | 2 |
| Commercial kits used | Invitrogen | Qiagen | Other |
|  | UltraSense/SuperScriptIII | QuantiTect Probe/MP/One-Step RT PCR |  |
| n laboratories | 2/3 | 2/1/2 | 5 |
| n laboratories with discordant results | 0/3 | 2/1/2 | 5 |

Invitrogen UltraSense: Invitrogen RNA UltraSense™ One-Step Quantitative RT-PCR System; Invitrogen SuperScriprIII: Invitrogen SuperScript® III One-Step RT-PCR System with Platinum®*Taq*; Qiagen QuantiTect Probe/MP: QIAGEN® QuantiTect Probe PCR Kit/ QIAGEN® QuantiTect Multiplex PCR Kit; Qiagen One-Step RT-PCR: QIAGEN® OneStep RT-PCR Kit
