# Supplementary material for: A Step Forward in Molecular Diagnostics of Lyssaviruses – Results of a Ring Trial among European Laboratories
Source: PLoS One. 2013 Mar 8;8(3):e58372. doi: 10.1371/journal.pone.0058372 (PMC3592807; doi:10.1371/journal.pone.0058372)
Supplement: Table S3 — Mean quantification cycle (Cq) values of a sample set testing before and after a freeze-thaw cycle (Freeze-thaw control). (DOC) [file pone.0058372.s003.doc]

**Table S3:** Mean quantification cycle (C_q_) values of a sample set testing before and after a freeze-thaw cycle (Freeze-thaw control)

|  |  | before freeze-thaw | | | | after freeze-thaw | | | |
| --- | --- | --- | --- | --- | --- | --- | --- | --- | --- |
| **sample** | **species** | **R13** | **R14** | **EBLV-1** | **EBLV-2** | **R13** | **R14** | **EBLV-1** | **EBLV-2** |
| **L-01** | RABV | 26 | 26 | N/A | N/A | 26 | 26 | N/A | N/A |
| **L-02** | RABV | 24 | 24 | N/A | N/A | 24 | 23 | N/A | N/A |
| **L-03** | RABV | 25 | 28 | N/A | N/A | 25 | 27 | N/A | N/A |
| **L-04** | RABV | 24 | 24 | N/A | N/A | 24 | 23 | N/A | N/A |
| **L-05** | RABV | 26 | 20 | N/A | N/A | 25 | 20 | N/A | N/A |
| **L-06** | RABV (I) | 27 | 27 | N/A | N/A | 27 | 26 | N/A | N/A |
| **L-07** | neg | N/A | N/A | N/A | N/A | N/A | N/A | N/A | N/A |
| **L-08** | RABV | 26 | 25 | N/A | N/A | 25 | 24 | N/A | N/A |
| **L-09** | RABV | 19 | N/A | N/A | N/A | 19 | N/A | N/A | N/A |
| **L-10** | RABV | 23 | 26 | N/A | N/A | 23 | 26 | N/A | N/A |
| **L-11** | RABV | 25 | 24 | N/A | N/A | 25 | 24 | N/A | N/A |
| **L-12** | RABV | 19 | 14 | N/A | N/A | 18 | 13 | N/A | N/A |
| **L-13** | RABV | 18 | 18 | N/A | N/A | 17 | 17 | N/A | N/A |
| **L-14** | RABV | 22 | 21 | N/A | N/A | 22 | 21 | N/A | N/A |
| **L-15** | RABV | 27 | N/A | N/A | N/A | 26 | N/A | N/A | N/A |
| **L-16** | RABV | 25 | 25 | N/A | N/A | 24 | 25 | N/A | N/A |
| **L-17** | RABV | 21 | 19 | N/A | N/A | 22 | 19 | N/A | N/A |
| **L-18** | EBLV-1 | N/A | N/A | 21 | N/A | N/A | N/A | 20 | N/A |
| **L-19** | RABV | 25 | 24 | N/A | N/A | 24 | 24 | N/A | N/A |
| **L-20** | RABV | 20 | 19 | N/A | N/A | 19 | 18 | N/A | N/A |
| **L-21** | RABV | 21 | 26 | N/A | N/A | 21 | 24 | N/A | N/A |
| **L-22** | RABV | 26 | 25 | N/A | N/A | 26 | 25 | N/A | N/A |
| **L-23** | RABV | 18 | 18 | N/A | N/A | 18 | 18 | N/A | N/A |
| **L-24** | EBLV-2 | N/A | N/A | N/A | 18 | N/A | N/A | N/A | 18 |
| **L-25** | neg | N/A | N/A | N/A | N/A | N/A | N/A | N/A | N/A |
| **L-26** | RABV | 32 | 27 | N/A | N/A | 31 | 26 | N/A | N/A |
| **L-27** | RABV (II) | 31 | 30 | N/A | N/A | 30 | 30 | N/A | N/A |
| **L-28** | RABV (III) | 34 | 33 | N/A | N/A | 34 | 33 | N/A | N/A |
| **L-29** | RABV | N/A | 17 | N/A | N/A | N/A | 17 | N/A | N/A |
| **L-30** | RABV (0) | 24 | 24 | N/A | N/A | 24 | 23 | N/A | N/A |

RABV: *Rabies virus*; EBLV: *European Bat Lyssavirus*; no C_q_: no C_q_ value available; R13, R14: R13, R14 assay by [17]; EBLV-1, EBLV-2: Freuling unpublished
