# Supplementary material for: A Step Forward in Molecular Diagnostics of Lyssaviruses – Results of a Ring Trial among European Laboratories
Source: PLoS One. 2013 Mar 8;8(3):e58372. doi: 10.1371/journal.pone.0058372 (PMC3592807; doi:10.1371/journal.pone.0058372)
Supplement: Table S4 — Overview of the overall results of laboratories which performed more than one assay. (DOC) [file pone.0058372.s004.doc]

**Table S4:** Overview of the overall results of laboratories which performed more than one assay.

|  |  | **lab A** | | **lab D** | | **lab F** | | **lab I** | | **lab J** | | **lab N** | | **lab O** | | **lab P** | |
| --- | --- | --- | --- | --- | --- | --- | --- | --- | --- | --- | --- | --- | --- | --- | --- | --- | --- |
| **sample** | **species** | pos. | neg. | pos. | neg. | pos. | neg. | pos. | neg. | pos. | neg. | pos. | neg. | pos. | neg. | pos. | neg. |
| **L-01** | RABV | 2 | - | 3 | - | 2 | - | 5 | 1 | 2 | - | 2 | - | 2 | - | 2 | - |
| **L-02** | RABV | 2 | - | 3 | - | 2 | - | 6 | - | 2 | - | 2 | - | 2 | - | 2 | - |
| **L-03** | RABV | 2 | - | 2 | 1 | 1 | 1 | 6 | - | 1 | 1 | 2 | - | 1 | 1 | 2 | - |
| **L-04** | RABV | 2 | - | 2 | 1 | 2 | - | 6 | - | 2 | - | 2 | - | 2 | - | 2 | - |
| **L-05** | RABV | 1 | 1 | 2 | 1 | 2 | - | 5 | 1 | 2 | - | 1 | 1 | 2 | - | 2 | - |
| **L-06** | RABV (I) | 2 | - | 2 | 1 | 2 | - | 6 | - | 2 | - | 2 | - | 2 | - | 2 | - |
| **L-07** | neg | - | 2 | - | 3 | - | 2 | - | 6 | - | 2 | - | 2 | - | 2 | - | 2 |
| **L-08** | RABV | 2 | - | 3 | - | 2 | - | 6 | - | 2 | - | 2 | - | 2 | - | 2 | - |
| **L-09** | RABV | 1 | 1 | 3 | - | 1 | 1 | 5 | 1 | 1 | 1 | 2 | - | 2 | - | 1 | 1 |
| **L-10** | RABV | 2 | - | 3 | - | 1 | 1 | 6 | - | 1 | 1 | 2 | - | 1 | 1 | 2 | - |
| **L-11** | RABV | 2 | - | 3 | - | 2 | - | 6 | - | 2 | - | 2 | - | 2 | - | 2 | - |
| **L-12** | RABV | 1 | 1 | 2 | 1 | 2 | - | 5 | 1 | 2 | - | 1 | 1 | 2 | - | 2 | - |
| **L-13** | RABV | 2 | - | 3 | - | 2 | - | 6 | - | 2 | - | 2 | - | 2 | - | 2 | - |
| **L-14** | RABV | 2 | - | 2 | 1 | 2 | - | 6 | - | 2 | - | 2 | - | 2 | - | 2 | - |
| **L-15** | RABV | 1 | 1 | 3 | - | 1 | 1 | 4 | 2 | 1 | 1 | 2 | - | 2 | - | 1 | 1 |
| **L-16** | RABV | 2 | - | 3 | - | 2 | - | 6 | - | 2 | - | 2 | - | 2 | - | 2 | - |
| **L-17** | RABV | 2 | - | 2 | 1 | 2 | - | 5 | 1 | 2 | - | 2 | - | 2 | - | 2 | - |
| **L-18** | EBLV-1 | 1 | - | 2 | - | 1 | - | 5 | - | 2 | - | 2 | - | 2 | - | 1 | - |
| **L-19** | RABV | 2 | - | 3 | - | 2 | - | 5 | 1 | 2 | - | 2 | - | 2 | - | 2 | - |
| **L-20** | RABV | 2 | - | 3 | - | 2 | - | 6 | - | 2 | - | 2 | - | 2 | - | 2 | - |
| **L-21** | RABV | 2 | - | - | **3** | - | **2** | 5 | 1 | 1 | 1 | 2 | - | 1 | 1 | 2 | - |
| **L-22** | RABV | 2 | - | 3 | - | 2 | - | 6 | - | 2 | - | 2 | - | 2 | - | 2 | - |
| **L-23** | RABV | 2 | - | 3 | - | 2 | - | 6 | - | 2 | - | 2 | - | 2 | - | 2 | - |
| **L-24** | EBLV-2 | 1 | - | 1 | - | 1 | - | 5 | - | 2 | - | 2 | - | 2 | - | 1 | - |
| **L-25** | neg | - | 2 | - | 3 | - | 2 | - | 6 | 1 | 1 | - | 2 | - | 2 | - | 2 |
| **L-26** | RABV | 2 | - | 3 | - | 2 | - | 6 | - | 1 | 1 | 2 | - | 2 | - | 2 | - |
| **L-27** | RABV (II) | 2 | - | 2 | 1 | 2 | - | 6 | - | 2 | - | 2 | - | 2 | - | 2 | - |
| **L-28** | RABV (III) | 2 | - | 2 | 1 | 2 | - | 4 | 2 | - | **2** | - | **2** | 1 | 1 | 2 | - |
| **L-29** | RABV | 1 | 1 | 1 | 2 | 2 | - | 5 | 1 | 2 | - | 1 | 1 | 2 | - | 1 | 1 |
| **L-30** | RABV (0) | 2 | - | 2 | 1 | 2 | - | 5 | 1 | 2 | - | 2 | - | 2 | - | 2 | - |

pos.: number of positive results for the individual sample; neg.: number of negative results for the individual sample; false negative results in all tests used are indicated in bold
